# Genetic Aspects of Dental Impaction: A Scoping Review

**DOI:** 10.3390/genes17030265

**Published:** 2026-02-26

**Authors:** Elena Oliva-Ferrusola, María Baus-Domínguez, Daniel Torres-Lagares, Maria-Angeles Serrera-Figallo

**Affiliations:** Department of Dentistry, Faculty of Dentistry, University of Seville, 41009 Seville, Spain; mbaus95@gmail.com (M.B.-D.); maserrera@us.es (M.-A.S.-F.)

**Keywords:** gene, genetics, dental impaction, DNA mutations, scoping review

## Abstract

**Background/Objectives:** There is a lack of cohesion in integrating current knowledge on the genetic and environmental etiology of dental impaction. The primary aim of this article is to review the current literature to identify candidate genes involved in the pathogenesis of dental impaction. **Methods:** A scoping review was conducted following PRISMA-ScR guidelines to identify and organize the available body of evidence. Relevant literature was searched in MEDLINE (via PubMed), Scopus, and Web of Science, with the final search conducted on 03 January 2026. Eligibility criteria included case–control, cohort, cross-sectional observational, and case report studies in humans. Selected studies focused on syndromic and non-syndromic variants, inheritance patterns, and genetic analyses. Risk of bias was assessed using the Joanna Briggs Institute (JBI) Critical Appraisal Checklists and AMSTAR 2. **Results:** Only 18 studies met the eligibility criteria. Most articles were case reports and retrospective observational studies, revealing a multifaceted genetic landscape underlying dental impaction, with mutations affecting transcription factors and signaling pathways critical for odontogenesis, particularly *RUNX2*, *FGFR1*, *MSX1*, *PAX9*, and *AXIN2*. Overall, the included studies showed moderate methodological quality. **Conclusions:** Current evidence does not clearly support specific genes as causal factors in dental impaction, but instead suggests a complex, likely polygenic susceptibility that modulates the anatomical threshold for tooth eruption. This review highlights *RUNX2*, *FGFR1*, *MSX1*, *PAX9*, and *AXIN2*, as well as emerging candidates involved in eruption and bone remodeling pathways. Future progress depends on standardized phenotyping, large replicated cohorts, and functional studies linking genetic variation to dental follicle-mediated eruptive remodeling.

## 1. Introduction

The frequency of postoperative complications following impacted tooth extraction ranges from pain and inflammation to irreversible damage to noble anatomical structures [1]. Their frequency ranges from 4.6% to 30.9% of interventions, with prevalence increasing in direct proportion to the patient’s age [2]. Currently, it is still not possible to predict which teeth will remain impacted, as the etiology of this phenomenon has not been fully clarified. Although local factors were long considered to be primarily responsible for impaction, it is now recognized that genetics plays a decisive role in regulating eruption [3].

The prevalence of dental impact is increasing in the current population [4]. Various studies have shown that the expression of specific genes associated with odontogenesis, bone remodeling, and cell differentiation plays an essential role in tooth eruption [5]. Several studies have documented families in which eruption patterns are heritable, observing that specific polymorphisms can lead to primary eruption failure, agenesis, or dental impaction [6]. Thus, the study of the genome in dental impaction has gained relevance in recent years, as the molecular mechanisms that regulate odontogenesis have been better understood [7,8]. Tooth formation is a highly complex process, controlled by precise signals between epithelial and mesenchymal cells and regulated by a series of genes that determine the position, number, shape, and chronology of the teeth [9,10]. Mutations in these genes lead to anomalies in tooth development, including inclusions, defined as the absence of a tooth in the dental arch at the expected time, surrounded by bone, partially or entirely, without any apparent mechanical cause [11].

Among the most studied genes in odontogenesis are those involved in enamel formation (amelogenesis), such as *AMELX*, *ENAM*, *MMP20*, and *KLK4*, whose mutations can alter dental morphology and, in turn, indirectly influence the eruptive process [12]. Similarly, the *DSPP* gene, which is involved in dentinogenesis, has been linked to dental dysplasias that affect the tooth’s internal structure and can interfere with its normal eruption. Multiple genetic syndromes are associated with impacted supernumerary teeth and various inclusions, including alterations in genes such as *RUNX2* (cleidocranial dysplasia, Gardner syndrome, familial adenomatous polyposis, Rubinstein-Taybi syndrome) [13].

On the other hand, morphogenetic regulatory genes, especially those of the homeobox group such as *MSX1*, *PAX9*, and *AXIN2*, have been described as playing a crucial role in the specification of tooth type and position [14]. Mutations in these genes have been associated not only with tooth agenesis but also with alterations in the eruptive trajectory, which can predispose to inclusions [15]. Likewise, genetic variants have been observed that can modify the expression of growth and signaling factors such as BMP4, SHH, and FGF, which are essential for cell migration and differentiation during tooth eruption [16]. Disruption of these pathways can create an unfavorable environment for eruption, even in the absence of physical obstacles.

The available scientific evidence, although still limited, is beginning to point to a possible link between specific genetic alterations and dental impaction. However, there is still no comprehensive review that integrates and unifies the contributions of existing studies. Hence, the main objective of this article is to review the current literature to map candidate genes involved in the pathogenesis of dental impaction and to grade their genetic credibility and functional support. The literature review will identify recurrent genes and genetic variants associated with dental impaction, thereby supporting the role of genetic factors in this phenomenon.

## 2. Materials and Methods

### 2.1. Protocol and Registration

A scoping review was conducted following the PRISMA-ScR [17] criteria, using a mapping approach to identify, characterize, and organize the available evidence on the relationship between genetics and tooth eruption in humans. Unlike a systematic review aimed at quantitative synthesis, our objective is not to estimate aggregate effects but to identify the existence of genetic inheritance, which genes and variants have been studied, with what designs, in those populations, and with what functional or biological support. The protocol is based on the classic methodological frameworks of Arksey & O’Malley and their critical extension by Levac et al. It adopts the recommendations of the Joanna Briggs Institute (JBI) Manual for scoping reviews. For the report, we will follow PRISMA-ScR (2018) [17], and the search documentation will comply with PRISMA-S and undergo PRESS review to maximize transparency and reproducibility. This methodological choice was selected due to the presence of genetic association studies, exome analyses, transcriptomic explorations, and heterogeneous reports, which make a broad and descriptive approach advisable. The protocol developed was previously registered in the Open Science Framework (OSF), a free digital platform with DOI: https://doi.org/10.17605/OSF.IO/2439P, https://archive.org/details/osf-registrations-2439p-v1 (accessed on 6 January 2026).

### 2.2. Databases and Search Strategy

The key question was designed according to the PCC rule [18]:

Participants: Subjects with radiologically diagnosed dental impaction (any tooth and age).

Concept: Studies that carry out a genetic evaluation, gene expression study, or genetic association study of polymorphisms involved in the etiology or predisposition to dental impaction.

Context: No restrictions on healthcare setting or geographical location.

Which genes or genetic variants have been associated with the development of dental impaction in humans, and how have they been identified in different clinical contexts?

The review began with a pilot search in MEDLINE/PubMed to assess sensitivity, identify synonyms, refine operators and fields, and evaluate ‘sentinel studies’. Three relevant sentinel studies addressing the relationship between genes and tooth inclusions were identified. Subsequently, the final search was conducted in the PubMed, Scopus, and Web of Science databases using controlled descriptors from the MeSH (Medical Subject Headings) thesaurus. This strategy standardized the terms used, ensuring greater precision in information retrieval by linking each concept to its corresponding MeSH term, regardless of the authors’ language variations across articles. Only the following MeSH terms were used: “Tooth, Impacted” [MeSH], “failure of tooth eruption, primary” [Supplementary Concept]), “tooth, impacted/embryology” [MeSH]), (“tooth, impacted/etiology” [MeSH]), (“tooth, impacted/genetics” [MeSH]), (“tooth, unerupted/embryology” [MeSH]), (“tooth, unerupted/etiology” [MeSH]), (“tooth, unerupted/genetics” [MeSH]) “Genetics”.

In MEDLINE, the following search string was used: (“failure of tooth eruption, primary” [Supplementary Concept]) OR (“tooth, impacted/embryology” [MeSH]) OR (“tooth, impacted/etiology” [MeSH]) OR (“tooth, impacted/genetics” [MeSH]) OR (“tooth, unerupted/embryology” [MeSH]) OR (“tooth, unerupted/etiology” [MeSH]) OR (“tooth, unerupted/genetics” [MeSH]). In Web of Science and Scopus, the following search string was applied: (“primary failure of tooth eruption” OR “tooth impacted” OR “tooth unerupted” OR “tooth eruption”) AND (genetics OR genetic OR gen). Search strategies were adapted to each database’s syntax. This methodological decision addressed the need to focus the search on key concepts that accurately captured the phenomenon under study, prioritizing specificity over breadth. A time and language filter was applied, including articles from 2000 onwards in Spanish and English.

### 2.3. Inclusion Criteria and Study Selection

To collect all evidence related to the genetic aspects of tooth impaction, case reports, clinical studies, clinical trials, comparative studies, journal articles, systematic reviews, and twin studies providing quantitative or qualitative data on genetic aspects in healthy patients or patients with genetic syndromes presenting multiple tooth impactions, gene expression studies, GWAS, bioinformatic analyses, and molecular genetics studies. Letters, conference abstracts, personal opinions, and gray literature were excluded. Priority was given to articles that performed a genome analysis (Table 1).

Studies that specifically analyzed dental impaction were included. For these, we based our definition on the dental impaction description from the Oral Surgery Treatise by Gay Escoda and Berini Aytés. Impaction is defined as the cessation of a tooth’s eruption caused either by a physical barrier (another tooth, bone, or soft tissues) along the eruption path that is detectable clinically or radiographically, or by an abnormal position of the tooth. If a physical barrier, abnormal position, or development cannot be identified as an explanation for the interruption of eruption of a tooth germ that has not yet appeared in the oral cavity, we refer to this as primary retention.

The search was initiated by including terms similar to tooth inclusion in order to conduct a comprehensive search and not overlook any articles that could be of interest, leading to a subsequent manual selection step. In this step, studies that analyzed ectopic eruption, primary eruption failure, and impaction related to space were excluded because these are entities with their own characteristics that could bias the final objective of the review.

The literature search was conducted using ZOTERO 7.0.32 (Corporation for Digital Scholarship, Vienna, VA, USA) to record the review process. Screening began with the title and abstract. Two independent reviewers (E.O.F and D.T.L) evaluated the titles and abstracts of the identified studies using the previously defined inclusion and exclusion criteria.

Subsequently, a full-text review was conducted of the studies that met the criteria in the first phase. Their relevance was confirmed, and it was verified that they contained genetic data applicable to the objective of the review. Those that, despite an apparently relevant abstract, did not include specific genetic analyses or did not directly address the condition of dental impaction were excluded. In cases of persistent disagreement between reviewers, a third reviewer made the final decision (M.A.S.F). Agreement on the inclusion of articles by both evaluators was determined using Cohen’s kappa index (κ).

### 2.4. Data Collection

Two independent reviewers (E.O.F. and M.B.D.) performed the data extraction. The data extracted were title, study design, funding source, conflict of interest, study population, mean age and SD, %_women/men, N_total, follow-up in months, objective, main results, genetic analysis Yes/No, type of gene analysis, variant pathogenicity, risk of bias indicators, availability of full text, and reason for exclusion. Discrepancies between the two were resolved by a third senior reviewer (D.T.L).

In the absence of data, attempts were made to contact the corresponding authors by email. In vitro studies were excluded, as these methods.

Studies conducted on families and monozygotic twins were included, as they provide valuable information on the genetic inheritance of dental impaction. Articles with direct data on genes were categorized accordingly. The genetic factors to be analyzed will include: the specific genes or genetic variants studied, the type of genetic analysis performed, and the population studied. In other words, not only will the genetic studies themselves be evaluated, but also the presence of inheritance patterns of the inclusions.

### 2.5. Risk of Bias

Specific tools will be used to assess risk of bias according to the design of the included studies: the Joanna Briggs Institute (JBI) critical appraisal checklist [19] for case–control studies, cross-sectional studies, and case reports. The methodological quality and risk of bias of the included systematic reviews will be assessed using the AMSTAR-2 (A MeaSurement Tool to Assess Systematic Reviews) tool [20], widely used in the health sciences and recommended for reviews that include both randomized and non-randomized studies. The full details are available in the Supplementary Material.

## 3. Results

Initially, 3566 scientific articles were identified. After removing duplicates, 2339 remained and were examined by title, abstract, and full text, applying the inclusion and exclusion criteria outlined above. A total of 158 articles were selected for further review. Finally, 18 articles were included in the scoping review, using a checklist to verify full-text availability and eligibility. During this procedure, the two authors disagreed on 30 articles, resulting in an observed agreement (Po) of 91.5%, with a Cohen’s Kappa of 0.83, a high value indicating good agreement between the authors.

**Figure 1:**
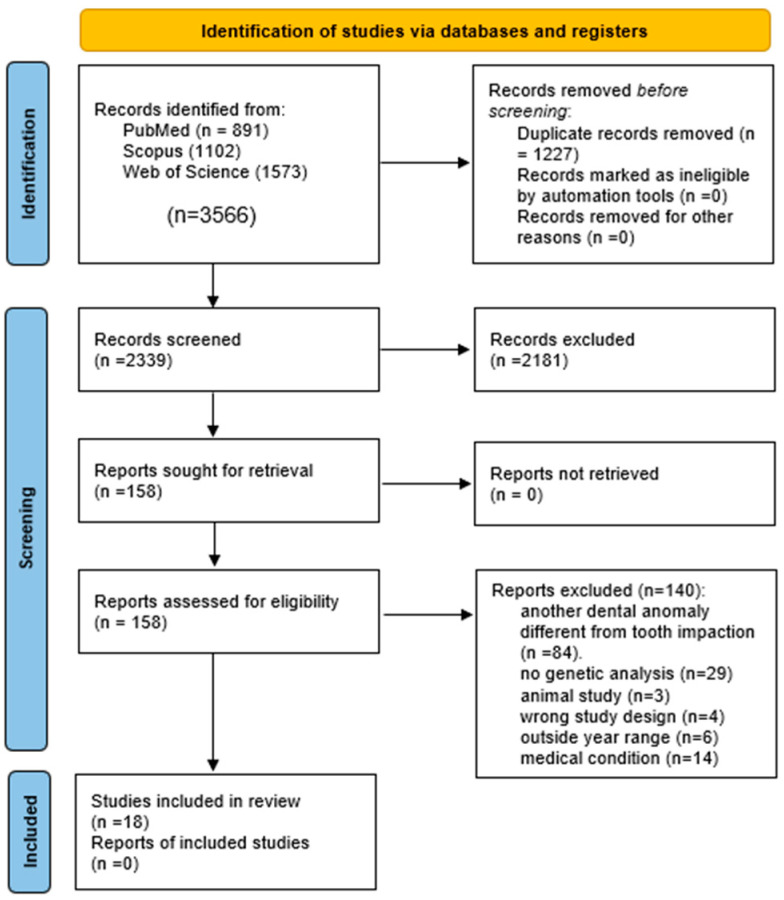
PRISMA 2020 flow diagram for PRISMA-ScR.

The review process from search to included articles is reflected in the PRISMA flow diagram (Figure 1).

### 3.1. Main Results

Finally, data extraction was performed for each article. Table 2 and Table 3 provide a summary of the primary data for quick comparison (study type, genetic analysis type, genetic variant, results, summary).

The set of studies included is heterogeneous in design and size: 7 case reports, 3 observational case–control studies, 6 experimental studies focused on gene expression in dental follicles/associated tissues, and 2 review papers. Studies in syndromic and non-syndromic populations have been added. The studies used various methodologies for genetic analysis, mainly whole-exome sequencing and real-time PCR (RT-PCR). A total of 1349 patients were studied (excluding reviews). In the syndrome studies, both primary and permanent teeth were included. In the non-syndromic studies, all dental groups (I, C, PM, M) and supernumerary teeth were included, with third molars (5 studies) and upper canines (6 studies) being the most numerous (Table 4).

The genes identified in the literature show varying levels of strength in the available evidence, which motivated their stratification according to the type of design and degree of causal inference. First, genes with strong evidence of monogenic or syndromic causality were identified, supported by pathogenic variants with familial segregation or association with defined clinical entities, such as *FGFR1*, *EDARADD*, and *NSD1*, in which inclusion or retention of teeth is interpreted as part of the phenotypic spectrum. Second, in non-syndromic tooth inclusion, genes like *MSX1*, *PAX9*, *AXIN2*, *MSX2*, and *ARNT2* present associative evidence derived from case–control studies, mainly in the form of polymorphisms or haplotypes linked to a higher risk of inclusion; however, these findings should be interpreted as susceptibility markers and not as evidence of causal pathogenicity. Finally, various transcriptomic and gene expression studies in pericoronal or follicular tissues have identified alterations in genes related to bone remodeling, mineralization, and inflammation—including *RUNX2*, *RANKL/OPG*, *SP7*, *BGLAP*, *IFITM5*, and immune mediators—as well as non-coding regulators. Nonetheless, these data represent contextual functional signals and do not allow for establishing direct germline causality. This stratification enables interpretation of the findings based on the strength of the evidence and avoids the implicit equivalence between methodologically different designs.

#### 3.1.1. Syndromic Dental Impactions and Genetic Associations

In the syndromic field, direct associations are shown between pathogenic variants and complex phenotypes involving multiple teeth. The most frequently associated syndromes were cleidocranial dysplasia, osteoglophic dysplasia, and Sotos syndrome. Two studies focus on cleidocranial dysplasia (CCD) and confirm the central role of *RUNX2*: a case report identifies an Alu-mediated microdeletion affecting *RUNX2* and *SUPT3H* [22], and a clinical-genetic study in 11 patients with CCD describes repeated oral features and genetic findings in *RUNX2* [23]. The syndromic evidence for *FGFR1* is also consistent. A case with 4 years of follow-up documents abnormal eruption and tooth retention in the context of osteoglophonyc dysplasia associated with a heterozygous mutation in *FGFR1* [24]. Similarly, another study proposes that a heterozygous mutation in *FGFR1* may be responsible for an incomplete form of the condition, characterized mainly by radiolucent bone lesions and dental retention [25]. Similarly, a case of impacted maxillary second molars in a patient with Sotos syndrome due to an *NSD1* mutation is presented [26].

#### 3.1.2. Non-Syndromic Dental Impactions and Genetic Associations

##### Genetic Variants

In the non-syndromic population, evidence of association is concentrated in maxillary canine impaction. Two case–control studies analyzed polymorphisms and haplotypes in *MSX1* and *PAX9*. They reported differences in haplotype distribution between subjects with impacted teeth and controls, with a signal of association for rs12532 (*MSX1*) and rs2073247 (*PAX9*) in an analysis including palatal impaction [27,28]. In contrast, a targeted genotyping study of *PAX9* found no statistically significant association between the variants analyzed and maxillary canine impaction [29]. Another survey of deeply impacted maxillary third molars found differential expression of *MSX1* [30]. On the other hand, the familial approach using sequencing provides exploratory evidence of candidate genes outside the *MSX1*/*PAX9* axis. Two exome studies in families with eruptive anomalies and normal karyotypes suggest a relationship with genes and familial aggregation without conclusive gene-variant assignment [31,32].

##### Molecular Mechanisms

Beyond DNA analysis, several studies explore local molecular mechanisms in the dental follicle. In impacted canine follicles, a comparative study reported changes in the expression of genes involved in bone remodeling and osteoclastic/osteoblastic signaling [33]. In impacted mandibular third molars, a survey of non-coding RNA regulation found increased *MEG3* and decreased *NORAD* in dental follicles compared to control tissue [34]. Another h y study analyzed ultra-conserved transcripts (T-UCRs) and showed differential expression of several T-UCRs in the follicular tissue of impacted mandibular molars [35]. Finally, a transcriptomic study of gingival tissues associated with impacted third molars reports large-scale alterations in gene expression [36].

##### Reviews

Finally, two synthesis studies are included. A narrative review of the etiology of maxillary canine impaction and a clinical-radiographic study proposing the sequential impaction hypothesis help integrate local developmental factors with biological susceptibility [37,38]. A systematic review focusing on the genetic basis of dental impaction identifies *MSX1*, *PAX9*, and *AXIN2* (among others) as recurrent candidates [39].

**Figure 2:**
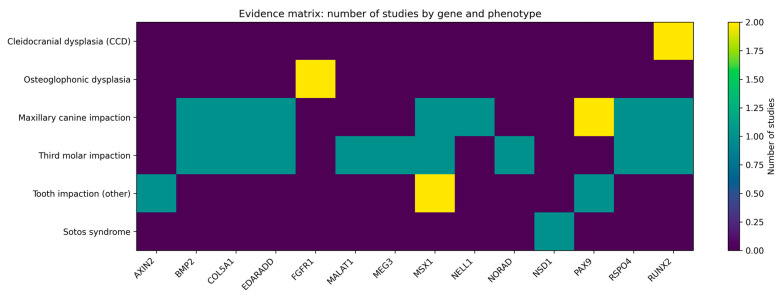
An evidence matrix summarizing the number of studies that have evaluated the association between candidate genes and different phenotypes related to dental impaction. Rows represent clinical phenotypes (isolated dental impaction, specific types of impacted teeth, and associated syndromes), while columns correspond to the genes analyzed. The color scale indicates the number of studies available for each gene-phenotype combination, ranging from 0 (no evidence) to 2. The synthesis matrix counts reported associations and should not be interpreted as an indicator of effect size or strength of evidence.

Figure 2 shows a summary image of the association between genes and phenotypes.

### 3.2. Risk of Bias Assessment

**Figure 3:**
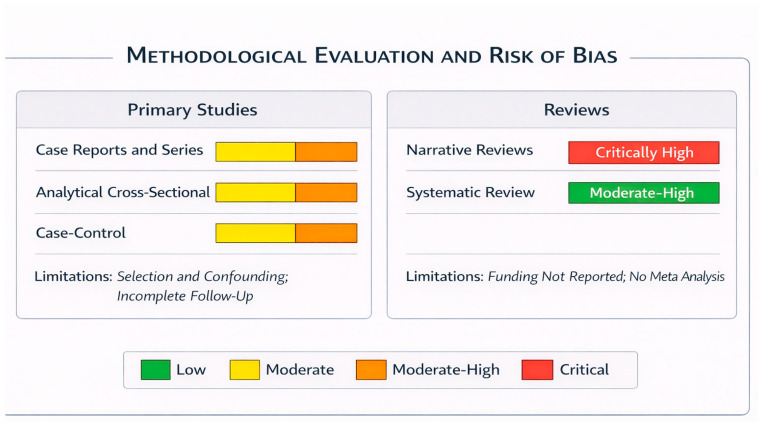
Methodological quality and risk of bias assessment of the included studies (JBI, AMSTAR 2). Primary studies (case reports and series, analytical cross-sectional studies, and case–control studies) were mainly assessed as having moderate to moderate–high risk of bias, primarily due to selection bias, confounding, and incomplete follow-up. Narrative reviews were classified as having a critically high risk of bias. In contrast, the systematic review showed a moderate-to-high risk, mainly due to the absence of meta-analysis and incomplete reporting of funding sources. Colors indicate the level of risk of bias, ranging from low (green) to critical (red).

The assessment of baseline and relative risk was performed using the classic checklist (JBI) for evaluating case–control studies and cross-sectional studies. Most of the included studies present a moderate overall risk of bias, with variations depending on the design. Genetic studies (*MSX1*, *PAX9*, and haplotypes) point to associations that are likely overestimated due to phenotype mixing (grouping “any impacted tooth”). Reports and case series, which predominate in the syndromic evidence, have inherent limitations, including poor generalizability, the absence of a control group, and incomplete clinical follow-up. Cross-sectional and case–control observational studies achieved intermediate scores, with small sample sizes, incomplete control of confounding, and, in tissue studies, non-equivalent controls as the primary sources of bias. Evidence from family series with sequencing was limited in applicability, and one study could not be adequately evaluated due to insufficient methodological information. In the reviews, AMSTAR 2 showed high–moderate confidence for the only systematic review included, while narrative reviews had critically low confidence. Overall, the available evidence is methodologically moderate (Figure 3).

As this study is a scoping review, an assessment of the risk of bias of the included studies was conducted to provide an overview of the methodological quality of the available evidence. However, consistent with the exploratory nature of scoping reviews, the risk-of-bias assessment results were not used to weight or exclude studies. They did not influence the interpretation of the findings or the study’s conclusions.

## 4. Discussion

### 4.1. Overall Assessment and Limitations

Dental impaction encompasses clinical entities with different mechanisms: locally caused dental impaction; multiple inclusions due to cranioskeletal syndromes; and primary eruption failure, which has a specific genetic etiology [5,37,38,40,41]. The presence of phenotypic variants helps explain some of the inconsistencies in studies of impacted teeth, since the genetic determinants of eruption arrest are not necessarily the same as those of ectopic eruption or impactions due to lack of space.

A rigorous analysis of the articles indicates that current studies on the genetic basis of dental impaction must be interpreted with caution. The data reveal a multifaceted genetic landscape underlying tooth impaction. The different studies expose both syndromic and non-syndromic forms and are intertwined with mutations in transcription factors and signaling pathways critical for odontogenesis.

In the non-syndromic studies collected, the focus on candidate genes (SNPs/haplotypes) predominates, with moderate sample sizes and vulnerability to known biases, including population stratification, inadequate control for multiple comparisons, and the absence of replication [27,28,29,39]. This limitation is compounded by variability in case definition and the lack of analysis of local characteristics that could act as confounding factors.

Molecular studies focusing on the dental follicle and the local microenvironment only allow new hypotheses to be generated due to their small samples; tissue heterogeneity (follicle vs. gum vs. peri-coronal tissues); sample chronology (relatively early vs. late pre-emergent stages); and confounding by inflammation, microflora, or post-surgical status [30,31,33,34,35,36].

In terms of design, most studies analyzing genetics are case reports [22,23,24,25,26,31,32]. A relevant limitation of the available evidence is the predominance of small-sample studies, particularly case reports, which account for a substantial proportion of the included studies (7/18). Although case reports are valuable for describing unusual clinical phenomena and generating preliminary hypotheses, they lack the methodological rigor required to establish robust associations or to estimate the magnitude of observed effects. Because they are based on individual cases or small case series, these studies are especially prone to selection bias, do not adequately control for confounding variables, and do not allow for the estimation of prevalence or the strength of associations. Consequently, while they provide important exploratory insights, their interpretative weight is limited, and their findings require confirmation through larger, well-designed analytical studies.

Similarly, the articles included analysis of the genome using a wide variety of techniques: Affymetrix, RTqPCR, whole exome sequencing (WES), GWAS, PCR, etc. This adds little information and significant variability that does not allow for comparison and limits the possibility of establishing consistent conclusions. Each technique analyzes different levels of sensitivity, coverage, and interpretation criteria, resulting in heterogeneous, incomparable results. For example, reliance on whole exome sequencing (WES) in isolated subjects, although valuable for identifying rare variants, does not capture complex polygenic interactions or environmental effects in large cohorts. In addition, many articles lack longitudinal controls, limiting understanding of how impacts evolve, and focus on specific populations (e.g., Asian or European), ignoring ethnic diversity that could reveal unique allelic variants. This methodological variability prevents the identification of common genetic patterns.

### 4.2. Syndromic Evidence

Regarding the analysis of impacts linked to genetic syndromes, dental impacts are presented in cleidocranial dysplasia and OGD syndrome. Studies confirm the association of *RUNX2* in cleidocranial dysplasia, where heterozygous variants such as c.674G > A or c.873_874delCA disrupt osteoblast differentiation and lead to multiple impacted teeth, as observed in familial cases [42]. This aligns with *RUNX2*’s role in regulating bone and tooth mineralization, suggesting that its dysregulation triggers a cascade of effects on tooth follicle maturation and eruption guidance [43]. For its part, the series of 11 patients with genetic analysis illustrates phenotypic variability among individuals affected by the same molecular axis [23].

In osteoglossal dysplasia, the included studies support the relationship between heterozygous *FGFR1* mutations and tooth retention, often accompanied by bone lesions and skeletal abnormalities [24,25]. Given that *FGF/FGFR* participates in craniofacial morphogenesis and bone homeostasis, it is plausible that its alteration modifies the dynamics of the eruptive microenvironment [25].

Similarly, a case of Sotos syndrome with mutations in *NSD1*, such as c.5020T > G, correlates with hypodontia and molar impaction, linked to altered craniofacial proportions [26]. *NSD1* is a global epigenetic regulator, so that dental manifestations may be pleiotropic, variable, and modulated by overall development.

Taken together, syndromic evidence indicates that the presence of multiple inclusions is associated with a high probability of syndromic etiology driven by alterations in genes related to bone metabolism. The integration of the genetic knowledge previously described in syndromic dental impactions is essential to advance our understanding of the genetic basis of non-syndromic dental impactions. In this context, analyzing these genes in non-syndromic individuals could identify genetic variants with subtle effects or incomplete penetrance, thereby clarifying the etiological heterogeneity of isolated dental impactions.

### 4.3. Nonsyndromic Variants and Signaling Pathways

In the non-syndromic spectrum, the study of classic odontogenesis genes stands out: *MSX1*, *PAX9*, and *AXIN2*, which are essential transcription factors in epithelial–mesenchymal interaction, dentition pattern, and dental development. These studies describe associations of variants with impacted teeth [27,29,44]. However, these are real but small signals, population-dependent, and in need of replication.

The study of the complete exome of families [32] suggests rare variants in genes linked to ectodermal development (*EDARADD*), extracellular matrix (*COL5A1*), Wnt potentiation (*RSPO4*), and craniofacial morphogenesis (*NELL1*). These candidate genes suggest that impaction results from the sum of variations in development and timing; tooth shape and size; biomechanics of the environment; and bone remodeling. In particular, *RSPO4* and *AXIN2* suggest a role for Wnt in susceptibility, while *COL5A1/NELL1* points to properties of the supporting tissue and to morphogenesis. Similarly, the etiological review of the maxillary canine [37] provides frameworks in which local factors clearly play a role. Genetics can modify the threshold of vulnerability.

In addition, the analysis of family cases in non-syndromic individuals [31] provides relevant evidence of autosomal dominant transmission of the trait, based on a pattern of vertical inheritance independent of sex. This type of transmission, observed consistently across generations, reinforces the hypothesis of an underlying genetic basis, as the persistence of the phenotype from parents to children suggests a heritable effect that transcends the influence of shared environmental factors.

### 4.4. Epigenetic Regulation and Emerging Evidence

Tooth eruption requires a dental follicle that regulates bone resorption and apposition. As summarized by Wise [44], this process depends on spatial and temporal changes in follicular gene expression, mediated by pathways such as RANKL/OPG, CSF-1, and VEGF, which are essential for the eruptive phase of bone remodeling. However, studies in human canines show that, in pre-emergent stages, the follicle exhibits high expression of osteogenic markers and low osteoclastic recruitment signals, with no apparent differences between impacted canines and controls [33]. Consistent with this, the differential expression of *MSX1* described in deeply impacted maxillary third molars supports the existence of expression programs associated with dental depth and position [30]. Taken together, these findings reinforce the hypothesis that tooth impaction results from a dynamic interaction between position, time, and molecular regulation of the follicle.

Studies on non-coding RNA [34] and differential T-UCRs [35] broaden the picture to post-transcriptional regulation. These epigenetic regulators could act as a link between genetic variants and phenotypic variability, explaining why similar mutations can have different effects across individuals [45]. Even so, these studies should be interpreted with caution, as the pericoronal tissue of third molars may capture an inflammatory or reparative response, making it essential to analyze these data within the framework of “tissue response” rather than as primary determinants of impaction.

### 4.5. Clinical and Research Implications

Hypothetically, the interaction between these previously described genes could form a polygenic risk model for impaction, where *MSX1/PAX9* variants act additively with *RUNX2* to exacerbate eruption failures in non-syndromic cases, coupled with a notion supported by the observation of identical phenotypes in twins and families suggesting heritable modifiers [46,47] A 2023 study [48] analyzes the genotype-phenotype of selective dental eruption failures, linking mutations in *PTH1R* to specific patterns of failed eruption, recommending a classification based on causal genes to improve diagnosis.

Compared with other dental anomalies, the advances proposed here, such as the use of GWAS to redefine polygenic risk, resemble those achieved in other areas of dentistry. In tooth agenesis, GWAS studies identified loci in *EDA* and *WNT10A*, leading to early diagnosis and personalized therapies, similar to what could occur with impactions [49,50]. Similarly, in the case of cleft lip and palate, advances in mass sequencing have enabled the development of polygenic risk scores that guide early prevention strategies [51].

A similar model could be applied to dental impactions to anticipate eruption failures from the embryonic stage. This parallel suggests that the field of dental impaction could move toward more robust and personalized clinical predictions. For example, genetic testing for variants in key genes (*MSX1*, *PAX9*, *AXIN2*, etc.) could identify high-risk patients during pediatric consultations, enabling early radiographic monitoring and preventive orthodontic planning. This would allow orthodontic treatment to begin in cases where key teeth—such as permanent canines—are expected to have insufficient space to erupt properly, thus avoiding complications such as occlusal guidance, impaction, or more complex tooth replacements in the future [52]. As highlighted in clinical studies on orthopedic correction of skeletal Class II malocclusions in growing patients, dentofacial development is highly responsive to biomechanical modulation during growth. This underscores that genetic susceptibility may interact with functional and skeletal factors, suggesting that impaction should be interpreted within a multifactorial model integrating molecular, developmental, and biomechanical influences [53]. A 2025 review [54] supports this statement, advocating planning that includes genetic testing to develop appropriate preventive treatment plans that enable effective management of patients’ symptoms.

The initial step in these genetic tests is identifying candidate genes that cause dental impaction. To consolidate the emerging evidence, future studies should begin with a phenotypic definition and standardization to separate impaction due to guidance/space from PFE and other disorders. It is essential to standardize location (palatal vs. vestibular), severity (depth), unilateralism, and chronology, and to define multicenter cohorts and multi-population replication. Polygene genetic testing may provide a more comprehensive view than isolated SNP testing [36]. Due to the current preponderance of small studies, case reports, and non-replicated associations, these approaches should be considered exploratory. Genetic studies could be coupled with functional models that test effects on follicular signaling and remodeling, with confounding factors controlled for (inflammation, tissue, and stage) and findings validated and translated into functional hypotheses [55].

## 5. Conclusions

In conclusion, there is currently no clear evidence confirming the genes involved in dental impactions. Evidence suggests that genetics contribute a complex susceptibility, probably polygenic, modulating the anatomical threshold. This review presents the existing knowledge on genes such as *RUNX2*, *FGFR1*, *MSX1*, *PAX9*, and *AXIN2*, as well as emerging genes such as *COL5A* and *NELL*, in dental impactions. The integration of genes associated with syndromic dental impactions is a key approach to understanding the genetic basis of non-syndromic dental impactions, as these genes participate in standard biological processes involved in tooth eruption and bone remodeling. Advances in this field depend on standardized phenotyping, large replication cohorts, and a functional agenda that connects variants and regulation to the central role of the dental follicle in eruptive bone remodeling, enabling more accurate clarification of the roles of genetic determinants in dental impaction. In the future, integrating whole exome sequencing (WES) data into large cohorts could map genetic interactions, create polygenic risk scores (PRS) specific to impaction, and pave the way for genomic dentistry to prevent and treat dental impaction.

## Figures and Tables

**Table 1:** Inclusion and exclusion criteria.

Inclusion Criteria	Exclusion Criteria
Original research studies	Letters to the editor, editorials, conference abstracts without full articles, and personal opinions.
Studies in humans	Purely clinical or radiographic studies without genetic or molecular analysis.
Studies involving individuals diagnosed with impacted teeth in adults or children.	Studies in animals.
Genetic or molecular evaluation studies.	Studies that analyze different dental anomalies without specifically mentioning dental impaction.
Analysis of genetic polymorphisms (SNPs), gene expression, mutations, candidate genes, etc.	Studies that do not evaluate genes, gene expression, or genetic variants.

**Table 2:** Studies in syndromic populations classified by author, summary of genes analyzed, and method used.

Author	Study Design	Type of Variant	Type of Genetic Analysis
Yunzhu Qian et al.	Case report	*RUNX2*, *SUPT3H*	Affymetrix CytoScan HD Array, Thermo Fisher Scientific, Santa Clara, CA, USA and RTq PCR
Yuchun Zou et al.	Case report	*FGFR1* exon 9	WES
B. Olsson et al.	Experimental analytical study	*RUNX2*, *BMP2*, *MSX1*	qPCR
Oka, Ayaka et al.	Case report	*NSD1* (c.5020T > G,p.C1674G)	WES
Bufalino, A. et al.	Case series	R190Q;R225Q; R225Q; Q292fs → X299 *RUNX2*	Mutational analysis of the *RUNX2* gene using specific primers for eight coding exons and their splice junctions.
Pauline Marzin et al.	Case report	*FGFR1* (NM_023110.2): c.917C > T, p. Pro306Leu	WES

**Table 3:** Studies in non-syndromic populations classified by author, summary of genes analyzed, and method used.

Author	Study Design	Type of Variant	Type of Genetic Analysis
Trybek et al.	Cross-sectional case–control	rs12532 *MSX1*	qPCR. TaqMan MSX1 Assay, Applied Biosystems, Foster City, CA, USA.
Becker A et al.	Review	ND	ND
Uribe, P et al.	Observational analytical cross-sectional	*GUSB*, *RUNX2*, *CX43*, *OSX*, *ALP*, *OCN*, *BMP2*, *CSF-1*, *RANKL*, *OPG*, *MCP1*	Histological analysis + RTqPCR
Vitria, Evy et al.	Cross-sectional cohort study	rs375436662, rs12881240, rs4904210; *PAX9*	Amplification of *PAX9* exons 2, 3, and 4 by PCR and FASTQ analysis; identification of heterozygous SNPs visually by overlapping peaks in the chromatograms.
Ege, Bilal et al.	Comparative analytical experiment	*NORAD* and *MEG3*	qPCR
Bozgeyik et al.	Experimental, analytical	T-UCRs: uc.38, uc. 112, and uc.338	RT-qPCR
Inchingolo et al.	Case report	ND	G-banding technique (GTG) [21]
Anjana Devi et al.	Cross-sectional case–control study	*MSX1* rs12532 *PAX9* rs 2073247	PCR. TaqMan Assay, Applied Biosystems, Foster City, CA, USA
S. Papadopoulos et al.	Review	*MSX1* rs12532, *PAX9* rs4904210 and rs2073247, *AXIN2* rs2240308, *MSX2* rs4868444, and *ARNT2* rs140220410	Genome-wide association studies (GWAS), real-time polymerase chain reaction (RT-PCR), and others.
Sajnani et al.	Descriptive cross sectional observational	ND	ND
Haolin Zhou et al.	Observational case control study	UR: *HLA-DRB4*, *CCL20*, and *CXCL8* DR: *SPRR2B*, *CLDN17*, *LCE3D*, and *LCE3E* *IFITM5*, *BGLAP*, and *UGT2B17*	Transcriptomic study (RNA-seq) with validation by qRT-PCR
Barbato E et al.	Case series	*EDARADD* rs1146322542; *COL5A* rs61735045; *RSPO4* rs6140807 T rs117097130; *NELL1* rs141323787	WES

**Table 4:** Summary table of the number of patients and phenotype of inclusions per study.

Author	Number of Patients	Inclusion Phenotype
Yunzhu Qian et al.	1	55, 54, 53, 52, 51, 61, 62, 63, 64, 73, 74, 82, 83, 84, 18, 28, 38, 48, supernumeraries
Yuchun Zou et al.	1	Incisors, Canines, premolars, temporary, and deciduous
Trybek et al.	392	canine (17), 1st PMS (2), 2nd MS (3), 3° maxillary molar (218), SS (1), mandibular first premolar (8), mandibular second premolar (4), CI (1), 2nd inferior molar (1), 3rd mandibular molar (144), supernumerary incisor (1)
Bufalino, A. et al.	11	Incisor, premolar, 1st molar, 2nd Molar, 3rd molar, supernumerary
Uribe, P et al.	11	Canines, mesiodens
Vitria, Evy et al.	132	Canines
Pauline Marzin et al.	3	1st mandibular molar, 2nd mandibular molar
Ege, Bilal et al.	30	3rd mandibular molar
B. Olsson et al.	32	3rd mandibular molar
Bozgeyik et al.	42	3rd mandibular molar
Inchingolo et al.	4	2nd molar, 3rd molar, maxillary incisor, canines, incisors, Supernumeraries
Oka, Ayaka et al.	1	2nd maxillary molars
Anjana Devi et al.	100	Canines
Sajnani et al.	533	Canines
Haolin Zhou et al.	40	3rd molars
Barbato E et al.	14	Maxillary canines

## Data Availability

No new data were created or analyzed in this study.

## Supplementary Materials

The following supporting information can be downloaded at: https://www.mdpi.com/article/10.3390/genes17030265/s1.
