# Supplementary material for: Genetic Aspects of Dental Impaction: A Scoping Review
Source: Genes (Basel). 2026 Feb 26;17(3):265. doi: 10.3390/genes17030265 (PMC13025325; doi:10.3390/genes17030265)
Supplement: Supplementary file 1 [file genes-17-00265-s001.zip › RISK OF BIAS.pdf]

RISK OF BIAS

Table S1. Resultados JBI (1 línea por artículo)

**Orthodontic Management of Severe…**	Reporte de caso	JBI Case Report (8 ítems)	6/8	**Moderado**	Información incompleta sobre **eventos adversos**, y **follow-up**/línea temporal poco detallada.
**A novel Alu-mediated microdeletion in the RUNX2 gene…**	Reporte de caso	JBI Case Report (8)	7/8	Moderado–Bajo	Limitaciones típicas: **generalización** n=1; a veces faltan **eventos adversos**/alternativas diagnósticas exhaustivas.
**Heterozygous FGFR1 mutation may be responsible for an incomplete form of osteoglophonic dysplasia…**	Serie/reporte(s) de caso	JBI Case Report (8)	6/8	Moderado	**Selección** (casos raros), posible falta de **timeline** y de discusión de **alternativas**/adversos completa.
**Cleidocranial 11 patients..**	Serie de casos (11 pacientes)	JBI Case Series (10)	6/10	Moderado–Alto	Posible falta de **inclusión consecutiva/completa**, ausencia de **grupo control**, y control limitado de **confusión**.
**The sequential hypothesis of impaction of maxillary…**	Estudio retrospectivo (n=533)	JBI Analytical Cross-Sectional (8)*	5/8	Moderado	Riesgo de **sesgo de selección** (muestra clínica), **confusión** (anomalías dentales/edad/sexo) no siempre controlada, mediciones radiográficas retrospectivas.
**Gene expression profiles in dental follicles…**	Transversal analítico (muestras DF; comparación por situación clínica)	JBI Analytical Cross-Sectional (8)	6/8	Moderado	Tamaño muestral pequeño y **heterogeneidad**; confusión clínica (p. ej., variables de desarrollo) no siempre ajustada.
**Non-coding RNAs transcribed from…**	Transversal analítico (DF vs tejido gingival control)	JBI Analytical Cross-Sectional (8)	6/8	Moderado	Control tisular **no equivalente** (gingiva vs folículo), posible **confusión** por inflamación/biología del tejido; ajuste limitado.
**Investigation of the expression level…**	Transversal analítico (DF vs gingiva)	JBI Analytical Cross-Sectional (8)	5/8	Moderado–Alto	Control tisular **no equivalente**, confusión (sexo/posición/biología local) con control limitado; representatividad.

**MSX1 is differentially expressed…**	Transversal analítico (comparación por fenotipo/profundidad)	JBI Analytical Cross-Sectional (8)	5/8	Moderado	Confusión (fenotipos y clasificación), tamaños por subgrupo pequeños; generalización limitada.
**Genotyping analysis of the…**	Casos y controles	JBI Case-Control (10)	7/10	Moderado	Confusión (edad/sexo/etnicidad/hábitos) y control estadístico no siempre completo; riesgo de sesgo de selección.
**Role of polymorphisms of MSX1 and…**	Casos y controles (50/50)	JBI Case-Control (10)	7/10	Moderado	Confusión potencial (estratificación poblacional), ajuste limitado; definición/control de exposición genética OK.
**Association of Polymorphic and Haplotype…**	Casos y controles (IMTM vs controles)	JBI Case-Control (10)	7/10	Moderado	Confusión (población/ancestría), posible sesgo de selección; control parcial de confusores.
**Whole exome sequencing in an Italian family with isolated maxillary**	Serie familiar (WES en afectados)	JBI Case Series (10)	6/10	Moderado	**Selección** familiar, sin comparador externo; aplicabilidad limitada; posibilidad de confusión genética/poblacional.
**Abnormal eruption of teeth in relation…**	**No clasificable con seguridad** (texto insuficiente)	—	—	Incierto	El PDF recupera principalmente **referencias**, sin métodos/casos completos para aplicar checklist con validez.

2) AMSTAR 2 – Evaluación de revisiones (una línea por artículo)

Artículo (archivo)	Tipo de revisión	Items "Sí" (aprox.)	Debilidades clave (AMSTAR 2)	Confianza global AMSTAR 2
**The genetic bassis of tooth.pdf**	**Revisión sistemática** (con protocolo)	13/16	En general sólida: protocolo/criterios y evaluación RoB reportados. Limitaciones típicas: a veces **financiación de estudios incluidos** no detallada y/o publicación sesgo no aplicable si no hay meta-análisis.	**Alta–Moderada**
**5.Tooth_Eruption_Disorders_from_Delayed_Eruption_to_.pdf**	Revisión narrativa (con búsqueda, pero no SR formal)	4/16	Sin protocolo, sin selección/extracción por duplicado, sin lista de excluidos, sin RoB formal ni síntesis estructurada tipo SR.	**Críticamente baja**

**10.Etiology of maxillary canine impaction a review.pdf**	Revisión narrativa/conceptual	2/16	No métodos de búsqueda reproducibles; sin protocolo, sin RoB, sin selección/extracción por duplicado; enfoque conceptual.	**Críticamente baja**

Evaluación item por item de cada articulo.

JBI – Reportes de caso (CR, 8 ítems)

Artículo	Diseño	CR1 Demografía	CR2 Historia/cronología	CR3 Condición clínica	CR4 Diagnóstico	CR5 Intervención	CR6 Resultados/seguimiento	CR7 Eventos adversos	CR8 Lecciones
Orthodontic Management of Severe…	Reporte de caso	Cumple—edad/sexo y contexto	Parcial—historia y plan; línea temporal no completa	Cumple—hallazgos clínicos/oclusales detallados	Cumple—fotos, Rx/CBCT, cefalométrico	Cumple—plan, aparatología y pasos	Cumple—resultados y retención	Parcial—menciona riesgos (p.ej., reabsorción); no reporta EA sistemáticos	Cumple—discusión con mensajes clave
Abnormal eruption of teeth in relation (2).pdf	Reporte de caso	Cumple—edad/sexo; fenotipo	Parcial—seguimientos descritos; cronología parcial	Cumple—intra/extraoral detallado	Cumple—panorámica, CBCT, genética, DDx	Parcial—plan multidisciplinar; detalles técnicos limitados	Parcial—evolución clínica y estudios; sin resultados terapéuticos finales	No—no describe EA	Cumple—conclusiones y recomendaciones
A novel Alu-mediated microdeletion in the RUNX2 gene in a Chinese patient (2).pdf	Reporte caso (genético)	Cumple—21 años, sexo, antropometría	Parcial—historia familiar/curso breve	Cumple—rasgos CCD y dentición	Cumple—radiología + arrays/qPCR/Sanger	No—no intervención clínica reportada	Parcial—hallazgos genéticos; sin seguimiento clínico	No—no aplica/ no reporta	Cumple—implicación patogénica y mosaico

JBI – Series de casos (CS, 10 ítems)

**Artículo**	**Diseño**	**CS1 Criterios inclusión**	**CS2 Medición condición**	**CS3 Identificación condición**	**CS4 Consecutivo**	**CS5 Completo**	**CS6 Demografía**	**CS7 Clínica**	**CS8 Resultados/seguimiento**	**CS9 Contexto/centro**	**CS10 Estadística**
17.Cleidocranial 11 pa.pdf	Serie de casos (11 pacientes/5 familias)	Parcial—definidos por diagnóstico CCD; no criterios explícitos	Cumple—examen físico y radiográfico	Cumple—criterios clínicos + genética	No—no indica reclutamiento consecutivo	Parcial—describe 11; no aclara si todos elegibles incluidos	Parcial—edad/sexo por familia/caso; no tabla completa	Cumple—fenotipo oral y radiológico detallado	Parcial—resultados genéticos; sin seguimiento longitudinal	Cumple—universidad/servicio y país	No—sin análisis estadístico formal (descriptivo)
1-s2.0-S1769721219302708-main.pdf	Serie de casos (3 familiares)	Parcial—familia con lesiones óseas + retención dental; criterios no formalizados	Cumple—clínica + Rx + histología (NOF)	Cumple—WES + correlación fenotipo	No—familia; no consecutivo	Parcial—incluye 3; no aclara si hay más afectados/no evaluados	Cumple—edad/sexo y datos básicos por caso	Cumple—hallazgos óseos y dentales por caso	Parcial—curso clínico parcial; sin outcomes estandarizados	Cumple—hospital/servicios, país	No—no procede / descriptivo sin análisis
1-s2.0-S0003996918301201-main.pdf	Serie familiar/serie de casos (14 miembros; WES en 3)	Parcial—familia con anomalías caninas; criterios no explícitos	Cumple—evaluación clínica estandarizada	Cumple—WES + segregación Sanger	No—familia; no consecutivo	Parcial—evalúa 14 miembros; posible completo pero no lo declara	Parcial—datos por individuo resumidos; no siempre completos	Cumple—fenotipo canino detallado	Parcial—genética/segregación; seguimiento clínico limitado	Cumple—centros universitarios/hospitalarios	Parcial—pipeline bioinformático; sin estadística clínica

JBI – Transversales (Analytical Cross-Sectional, XS, 8 ítems)

Artículo	Diseño	XS1 Criterios inclusión	XS2 Sujetos/entorno	XS3 Exposición	XS4 Medición condición	XS5 Confusores	XS6 Control confusores	XS7 Resultados	XS8 Estadística
16.The sequential hypothesis of impaction of maxillary.pdf	Transversal retrospectivo (registros/radiografías)	Parcial—identificación por registros; criterios completos no listados	Cumple—n=533, periodo 1982-2009, radiografías descritas	Cumple—mediciones radiográficas estandarizadas	Parcial—clasifica bucal/palatina; criterio clínico-radiográfico, pero sin reproducibilidad	No—no identifica confusores relevantes (edad/sexo/otros) formalmente	No—sin ajuste; análisis principalmente descriptivo	Cumple—variables geométricas/edad dental con métodos descritos	Parcial—t pareada; no justifica supuestos ni múltiples comparaciones
MSX1 is differentially expressed.pdf	Transversal analítico (expresión génica vs fenotipo impacción)	Cumple—>18, ASA<III; excluye sindrómicos	Cumple—n=32, centro quirúrgico	Cumple—clasificación Winter/Pell&Gregory	Cumple—OPG + clasificación por experto	Parcial—menciona ASA/sindrómico; no otros (edad/sexo/medicación)	No—sin modelos multivariables	Cumple—qPCR en muestras óseas; métodos descritos	Cumple—Kruskal–Wallis/Dunn/Spearman apropiados
9.Gene expression profiles in dental follicles from patients.pdf	Transversal analítico (RT-qPCR; grupos clínicos)	Cumple—criterios/exclusiones descritos (síndromes, etc.)	Cumple—11 pacientes; muestreo en cirugía; controles descritos	Cumple—grupo: impactado con/sin reabsorción vs controles	Parcial—reabsorción por imagen; criterios no muy detallados	Parcial—excluye síndromes/metabólicos; no ajusta por edad/sexo/etapa	No—sin ajuste	Cumple—RT-qPCR siguiendo MIQE; genes referencia	Cumple—ANOVA para comparación; adecuado
3.Non-coding RNAs transcribed from.pdf	Transversal pareado (folículo vs encía en mismos pacientes)	Parcial—incluye 42; criterios clínicos no detallados del todo	Cumple—mar 2021–dic 2021; clínica OMFS; demografía en resultados	Cumple—tipo de tejido (folículo vs encía)	Cumple—impacción indicada por clínica; extracción quirúrgica	Parcial—evalúa demografía; otros no	Parcial—diseño pareado reduce confusión; sin análisis multivariable	Cumple—RT-qPCR con controles; métodos descritos	Cumple—t pareada/Wilcoxon; chi-cuadrado; adecuado

4.Investigation of the expression level.pdf	Transversal pareado (DF vs encía)	Parcial—30 con IMTM unilateral mesioangular; criterios resumidos	Cumple—pacientes quirúrgicos; tejidos definidos	Cumple—tipo de tejido	Cumple—IMTM asintomático sin radiolucencia patológica	Parcial—criterios radiológicos y asintomático; no otros	Parcial—pareado; sin ajuste adicional	Cumple—RT-qPCR; genes MEG3/NORAD etc	Parcial—comparaciones simples; no discute corrección múltiple

JBI – Casos y controles (CC, 10 ítems)

**Artículo**	**Diseño**	**CC1 Comparabilidad**	**CC2 Emparejamiento**	**CC3 Criterios casos/controles**	**CC4 Medición exposición**	**CC5 Igual medición**	**CC6 Confusores**	**CC7 Control confusores**	**CC8 Medición desenlace**	**CC9 Periodo exposición**	**CC10 Estadística**
7.Genotyping analysis of the.pdf	Casos y controles (PAX9 SNPs; 132 sujetos)	Parcial—edad/sexo en criterios; no compara otras variables	No—no empareja; solo criterios generales	Cumple—diagnóstico por clínica+Rx por expertos; criterios explícitos	Cumple—PCR + secuenciación; protocolos descritos	Cumple—misma técnica en ambos grupos	No—no identifica confusores (p.ej., etnia/edad/sexo/otros)	No—sin ajuste	Cumple—impacción confirmada por radiología	Cumple—genotipo antecede al desenlace	Parcial—OR/chi2; sin modelos multivariables ni corrección
8.Role of polymorphisms of MSX1 and.pdf	Casos y controles (50+50; emparejado)	Cumple—controles edad/sexo-matched; misma población	Cumple—emparejamiento por edad/sexo	Cumple—criterios incl/excl; diagnóstico Jacobs/CBCT	Cumple—PCR-RFLP para SNPs; método descrito	Cumple—misma genotipificación	Parcial—considera edad/sexo; no otros (p.ej., etnia subestruct.)	Parcial—emparejamiento; sin ajuste estadístico adicional	Cumple—impacción palatina confirmada	Cumple—genotipo	Cumple—OR + chi2 con IC95% (básico, adecuado)
11.Association of Polymorphic and	Casos y controles (genética; maxillary	Parcial—describe grupos; comparabilidad limitada	Parcial—posible matching parcial; no siempre claro	Cumple—definición de casos/controles y criterios	Cumple—genotipado (PCR/TaqMan/seq) descrito	Cumple—mismo método en ambos	Parcial—menciona sexo/edad; no otros	No—sin ajuste multivariable	Cumple—diagnóstico radiográfico/ortodóncico	Cumple—genotipo	Parcial—haplotipos/OR; reporte limitado de control de

Haplotype.pdf	canine impaction)										múltiples pruebas

AMSTAR 2 – Leyenda de ítems (I1–I16)

Tabla AMSTAR 2 (I1–I16)

**Revisión**	**Confianza**	**I1**	**I2**	**I3**	**I4**	**I5**	**I6**	**I7**	**I8**	**I9**	**I10**	**I11**	**I12**	**I13**	**I14**	**I15**	**I16**
The genetic basis of tooth impaction: a systematic review (2025)	Moderada–Alta	Sí — PICO/PECOS explícito.	Sí — protocolo registrado (PROSPERO CRD42024597963).	Sí — justifica diseños observacionales (genética) como criterio.	Sí — búsqueda en 10 bases; actualización a marzo 2025; términos/estrategia descritos.	Sí — selección por ≥2 revisores; acuerdo (Kappa 0.83).	Parcial — extracción menciona autores; no siempre explícito 'doble extracción' para todos los datos.	Parcial — flujo PRISMA y conteos; listado completo de excluidos con motivos no siempre exhaustivo.	Sí — descripción de estudios incluidos (diseño, genes, muestras).	Sí — RoB con checklists JBI para diseños incluidos; tablas RoB.	NC/Parcial — fuentes de financiación de estudios primarios no siempre detalladas.	N/A — sin meta-análisis (heterogeneidad).	N/A — sin meta-análisis.	Sí — discusión considera RoB/heterogeneidad y resultados contradictorios.	No/NC — evaluación formal de sesgo de publicación no aplicable/no realizada (sin MA).	N/A — sin análisis de heterogeneidad cuantitativo de MA; heterogeneidad cualitativa discutida.	Sí — conflictos/financiación declarados (sin financiación del estudio; OA HEAL-Link).

AMSTAR 2 (16 ítems)

**Artículo**	**Tipo revisión**	**1 PICO**	**2 Protocolo**	**3 Diseños inclusión**	**4 Búsqueda**	**5 Selección duplicada**	**6 Extracción duplicada**	**7 Excluidos**	**8 Detalle estudios**	**9 RoB**	**10 Financiación estudios**	**11 Meta-análisis**	**12 RoB en MA**	**13 RoB interpretación**	**14 Heterogeneidad**	**15 Sesgo publicación**	**16 COI/financiación revisión**

The genetic bassis of tooth.pdf	Sistemática (sin meta-análisis aparente)	Cumple—PECOS/PICO explícito	Parcial—menciona PRISMA/PECOS; PROSPERO/protocolo no completamente detallado	Cumple—observacionales especificados	Cumple—10 bases hasta mar-2025; estrategia descrita	Parcial—no siempre indica doble ciego/duplicado	Parcial—no claramente en duplicado	Parcial—puede haber diagrama; lista/justificación no completa	Cumple—tabla/descripcion de estudios	Cumple—JBI aplicado a estudios	No—no reporta fuentes de financiación de estudios primarios de forma sistemática	N/A—no meta-análisis	N/A—no meta-análisis	Cumple—discute RoB/limitaciones	Parcial—discute variabilidad; sin análisis formal	N/A—sin MA/ sin evaluación formal	Cumple—declara COI y/o financiación
10.Etiology of maxillary canine impaction a review.pdf	Narrativa	No—no pregunta estructurada	No—no	No—no define	No—sin estrategia reproducible	No—no	No—no	No—no	Parcial—discute literatura; sin tablas sistemáticas	No—no evalúa	No—no	N/A—no meta-análisis	N/A—no meta-análisis	Parcial—interpretación narrativa sin RoB formal	Parcial—discusión cualitativa	N/A—no meta-análisis	Parcial—COI/financiación puede estar; no siempre claro
5.Tooth_Eruption_Disorders_from_Delayed_Eruption_to_.pdf	Narrativa (JOHS 2025)	No—no pregunta PICO	No—no	No—no especifica	No—no describe búsqueda/criterios	No—no	No—no	No—no	Parcial—resumen general; sin detalle de cada estudio	No—no	No—no	N/A—no meta-análisis	N/A—no meta-análisis	Parcial—discute limitaciones generales	Parcial—discusión general	N/A—no meta-análisis	Parcial—puede declarar; revisar en PDF
